# Application of Gutta Percha to Pivot Teeth

**Published:** 1854-07

**Authors:** J. Richardson

**Affiliations:** Cincinnati


### *Application of Gutta Percha to Pivot Teeth.* By J RICHARDSON, M. D., D. D. S., Cincinnati.

I propose to describe briefly in this communication a method of inserting pivot teeth, the advantages of which, I think, will be apparent.

Gutta percha has already been moulded into many valuable appliances in general surgery, and we have good reason to believe that before the lapse of many years it will, in many important respects, be made available in dentistry. Experiments have already been made with it in this latter department of science, which, if they have not altogether succeeded, furnish at least sufficient motive for continued trial and reasonable assurances of ultimate success.

The following mode of applying it to pivot teeth, will at least commend itself for its *simplicity*, and not less I think for its *practicability* and *utility*.

After the root has been filed to its proper level, an impression of the front teeth may be taken, and from this a plaster model. To this plaster cast the pivot teeth can be more accurately ground, and in less time, than by trying it from time to time in the mouth. To economise time, the drilling of the fang may be completed while the plaster is hardening. A wood pivot should next be constructed, of a size that wil[illegible]

enable it to enter somewhat freely into the cavity of the tooth and fang. For the two ends of the pivot, a mixture of gutta percha and pumice stone may be used. This will render the pivot less likely to draw, which otherwise might occur, as it is inserted perfectly dry and designed to be kept so. In adjusting the pivot to the tooth, I fill the cavity of the latter part full with the mixture mentioned, and when it has become perfectly softened by holding it for a moment over the flame of a spirit lamp, the pivot is introduced and held there until it cools. It is then so firm that I have been unable to remove it, while cool, with a pair of pliers. At this stage, the tooth with the pivot should be again tried in the mouth, to see that the tooth when inserted will occupy the position you wish it; if not, the pivot should be carefully bent with the pliers until the tooth sits close and ranges with the other teeth. A very small strip of pure gutta percha sheeting, with its centre perforated, may then be slipped over the other end of the pivot on to the heel of the tooth, and the balance of the pivot smeared with a thin coating of the mixture. It is then ready for insertion; but as the pivot is more liable to draw from the fang than the tooth, it is well to roughen this end of the pivot before applying the gutta percha, and also to rim the cavity of the fang with a small, sharp-pointed excavator. I next dry the cavity and filed surface of the fang thoroughly, and placing a napkin closely to it, direct the patient to hold it there while I again heat the pivot tooth and its gum coating until the latter is quite soft. This may be allowed to cool, until it imparts but a slight sensation of warmth to the fingers, when it may be immediately introduced and pushed up with sufficient force to expel all superabundant gutta percha. The tooth should be properly adjusted while the material is still soft and pliable, otherwise its attachments will be endangered and its stability impaired. The expelled portions may now be trimmed off with a sharp pointed knife. This completes the operation, and so far as the application of gutta percha is concerned, it occupies much less time than has been consumed in its description. We have been somewhat full and particular in describing the mode of its application, because much depends upon proper manipulations, nor nave we been deterred by its seeming simplicity.

The advantages of this method are—

*First.* The cheapness of the material used, and the ease and facility with which the operation may be performed.

*Second.* It furnishes a uniform basis for the pivot tooth, so that any force applied to it will bear equally at all points, thus lessening the chances of any injurious strain upon the pivot or fang.

*Third.* Portions of gutta percha that are pressed out, may be made to fill up any decay in the fang that may happen to dip below the filed surface, thereby arresting further decay, and filling up what would otherwise furnish a receptacle for matter subject to decomposition.

*Fourth.* Pivots, which from the funnel-shaped conformation of many cavities, are rendered insecure by the ordinary method, will acquire increased stability by this.

*Fifth.* Its chief merit, however, consists, as will be apparent to all, in this, that it furnishes a complete barrier to the iutroduction of fluids into the cavity of the fang. This, in pivot teeth, is the paramount indication to be fulfilled, and we know of no method that has approached so nearly this "consummation devoutly to be wished," as the one we are presenting. Dr. Harris suggests a plan to effect this object, in his "Principles and Practice," but his mode is accompanied with so much expense and trouble, and is really so impracticable, that not one dentist in a hundred, we venture to affirm, ever resorts to it. We have tried the gutta percha in a number of instances, and are confirmed in the opinion that it has just claims to superiority, and that no artificial substitute can approach nearer the condition of the healthy natural organ than a pivot tooth properly inserted on this principle.

We offer it to the profession for what it is worth, believing at when fairly tested it will commend itself to their favor.

